# Augmented Liver Uptake of the Membrane Voltage Sensor Tetraphenylphosphonium Distinguishes Early Fibrosis in a Mouse Model

**DOI:** 10.3389/fphys.2021.676722

**Published:** 2021-10-25

**Authors:** Himanshi Pandita, Esteban Mezey, Shanmugasundaram Ganapathy-Kanniappan

**Affiliations:** ^1^Division of Interventional Radiology, Russell H. Morgan Department of Radiology and Radiological Science, The Johns Hopkins University School of Medicine, Baltimore, MD, United States; ^2^Division of Gastroenterology and Hepatology, Department of Medicine, The Johns Hopkins University School of Medicine, Baltimore, MD, United States

**Keywords:** liver fibrosis, membrane-voltage sensor, tetraphenylphosphonium (TPP), mitochondrial respiration, electron transport chain, carbon tetrachloride, liver voltage

## Abstract

Mitochondrial (mito-) oxidative phosphorylation (OxPhos) is a critical determinant of cellular membrane potential/voltage. Dysregulation of OxPhos is a biochemical signature of advanced liver fibrosis. However, less is known about the net voltage of the liver in fibrosis. In this study, using the radiolabeled [^3^H] voltage sensor, tetraphenylphosphonium (TPP), which depends on membrane potential for cellular uptake/accumulation, we determined the net voltage of the liver in a mouse model of carbon tetrachloride (CCl_4_)-induced hepatic fibrosis. We demonstrated that the liver uptake of ^3^H-TPP significantly increased at 4 weeks of CCl_4_-administration (6.07 ± 0.69% ID/g, *p* < 0.05) compared with 6 weeks (4.85 ± 1.47% ID/g) and the control (3.50 ± 0.22% ID/g). Analysis of the fibrosis, collagen synthesis, and deposition showed that the increased ^3^H-TPP uptake at 4 weeks corresponds to early fibrosis (F1), according to the METAVIR scoring system. Biodistribution data revealed that the ^3^H-TPP accumulation is significant in the fibrogenic liver but not in other tissues. Mechanistically, the augmentation of the liver uptake of ^3^H-TPP in early fibrosis concurred with the upregulation of mito-electron transport chain enzymes, a concomitant increase in mito-oxygen consumption, and the activation of the AMPK-signaling pathway. Collectively, our results indicate that mito-metabolic response to hepatic insult may underlie the net increase in the voltage of the liver in early fibrosis.

## Introduction

Liver fibrosis/cirrhosis represents a worldwide health problem, and epidemiological data indicate that 70–80% of cirrhotic patients develop primary liver cancer, hepatocellular carcinoma (Hernandez-Gea and Friedman, 2011). Liver fibrosis results from chronic inflammation and/or injury to the liver parenchyma. Irrespective of the cause, liver fibrosis may lead to cirrhosis and remains a major cause of liver failure (Farazi and DePinho, 2006; Amann et al., 2009).

Emerging reports underscore the role of mitochondrial (mito-) dynamics and alterations in mito-metabolism in liver diseases (Han et al., 2012; McCommis and Finck, 2019). Mito-dysfunction and structural abnormalities are the hallmarks of advanced liver fibrosis/cirrhosis (Krahenbuhl et al., 2000; Rodrigues and Steer, 2000; Grattagliano et al., 2011; Kang et al., 2016; Mansouri et al., 2018). Particularly, mito-oxidative phosphorylation (OxPhos) is impaired in the hepatocytes of advanced fibrosis and cirrhosis implying a switch in energy metabolism (Ganapathy-Kanniappan et al., 2014; Nishikawa et al., 2014). In contrast, in hepatic stellate cells (HSCs), the fibrogenic phenotype exhibits enhanced mito-bioenergetics, resulting in elevated mito-membrane potential (MMP, Δψ_m_) (Gajendiran et al., 2018). However, there is paucity in the documentation of net MMP of the liver in fibrogenesis. Noteworthy, OxPhos and the related electron transport chain (ETC) impact the mito-electrochemical gradient, a critical determinant of the net MMP. In this study, we investigated whether the mito-response/sensitivity to hepatic insult in early phases of fibrogenesis affects the net liver-MMP. Using the radiolabeled [^3^H] voltage sensor, tetraphenylphosphonium (TPP), which depends on membrane potential for cellular uptake/accumulation (Moreno et al., 2015), we determined the liver uptake of ^3^H-TPP in a mouse model of carbon tetrachloride (CCl_4_)-induced hepatic fibrosis.

Tetraphenylphosphonium and its analogs are extensively employed molecular probes in the analysis of MMP and the related bioenergetics (Kamo et al., 1979; Wan et al., 1993). In fact, one of the earliest reports, in the late 1960s, demonstrated the application of TPP as a chemical indicator of MMP (Liberman et al., 1969). The specificity and selectivity of the cellular TPP uptake and its correlation with MMP have been widely documented. Experimentally, in human skin fibroblasts, the cellular uptake of TPP correlates with the MMP, and a decrease in MMP reduces its cellular uptake (Rugolo and Lenaz, 1987). Besides, several TPP-conjugated therapeutic agents have also been shown to target cells with elevated mito-activity (Han et al., 2014; Pathak et al., 2014; Gajendiran et al., 2018). Thus, the mito-specificity of the TPP uptake has been well-established (Chen, 1988). Due to the relevance of mito-alterations in human pathophysiology, labeling TPP for application in positron emission tomography (PET) imaging is explored and successfully demonstrated in cardiac disease (Shoup et al., 2011; Gurm et al., 2012). Chemically, ^18^F-fluorophenyl-triphenylphosphonium is an analog of TPP with the substitution of fluorophenyl for one of the phenyl groups. Functionally, both TPP and ^18^F-triphenylphosphonium exhibit the lipophilic cationic property, and the affinity to accumulate within mitochondria depending on the MMP. Thus, radiolabeled [^3^H]-TPP and the ^18^F-triphenylphosphonium differ only in their imaging potential by PET (^18^F). Nevertheless, in hepatic fibrosis, there is a paucity in the experimental documentation of liver voltage, particularly in the early and advanced stages of progression.

Many factors including hepatotoxins are known to trigger progressive fibrotic disease of the liver (Wynn, 2008; Wynn and Ramalingam, 2012). Clinically, although less frequent, the intake of some therapeutics is known to cause hepatotoxicity and in some cases fibrogenesis (Zachariae et al., 1987; McDonnell and Braverman, 2006; Navarro and Senior, 2006; Han et al., 2013). Therefore, in this study, we intended to investigate the MMP (Δψ_m_) of global liver in hepatotoxin-induced fibrosis. The CCl_4_-induced liver-injury model is a well-established and widely used model of hepatotoxicity-induced fibrosis (Mehendale et al., 1994; Wang et al., 2007; Jin et al., 2011; Karthikeyan et al., 2016). As the objective of the model is to induce CCl_4_-based hepatotoxicity, we used olive oil (vehicle), a non-toxic, emulsifying agent that facilitates complete and homogenous mixing of CCl_4_. The control group animals received just the vehicle (olive oil). Thus, our objective was to determine if CCl_4_-induced hepatic fibrogenesis alters the MMP, net voltage of the liver. Furthermore, we evaluated its correlation with fibrogenesis and mito-respiration.

## Materials and Methods

### Radiolabeled [^3^H]-TPP

Tetraphenylphosphonium bromide, [phenyl-^3^H], i.e., ^3^H-TPP at a concentration of 37 MBq/ml was synthesized and supplied by the American Radiolabeled Chemicals Inc. (St Louis, MO, USA).

### Chemicals and Reagents

Unless otherwise mentioned, all chemicals were purchased from Sigma-Aldrich Co., (St. Louis, MO, USA). Primary antibodies such as AMPK β1 (#4150), phospho (p)-AMPK β1 (#4181), eIF2α (#9722), p-eIF2α (#3597), β-actin (#4970), mitoSTAT3 (#9139), and cytochrome C oxidase IV (COX IV; #4844) were from Cell Signaling Technologies Inc. (Danvers, MA, USA). Antibodies such as F_1_-F_0_ ATPase (5E) (#SC-81874) and mitochondrial-encoded cytochrome C oxidase subunit 1 (MT-CO1; #PA5-26688) were from Santa Cruz Biotechnology Inc. (CA, USA) and Thermo-Fisher Scientific Inc. (Grand Island, NY, USA), respectively. Secondary antibodies were purchased from Cell Signaling Technologies Inc. (#7076; #7174), Santa Cruz Biotechnology (#SC-2004), or Bio-Rad Laboratories (#170-6516) (Hercules, CA, USA). The alanine aminotransferase (ALT) assay kit to determine the liver function was procured from Bio Vision Inc. (San Francisco, CA, USA).

### Fibrogenesis by CCl_4_

Animal experiments were performed as approved by the Institutional Animal Care and Use Committee. To establish the liver fibrosis model, 3–4 week old male C57BL/6 mice (15–20 g body weight) were procured from the Charles River Laboratories Inc. (Wilmington, MA, USA) and maintained in a temperature-controlled room with an alternating 12-h dark and light cycle. To determine the fibrotic stage, mice were randomly divided into control (vehicle, *n* = 7) and experimental groups (*n* = 7) representing 2, 4, and 6 weeks of CCl_4_ administration. Fibrogenesis was induced by intraperitoneal administration of 20% solution of CCl_4_ (Sigma Chemical Co., St. Louis, MO, USA) in olive oil (vehicle) (Mehendale et al., 1994; Wang et al., 2007; Jin et al., 2011; Karthikeyan et al., 2016), at a dose of 0.5 μl/g bodyweight every week thrice for up to 6 weeks. Histopathology and analysis of fibrosis markers were used to determine the early fibrotic stage.

### Histopathology and Staging Fibrosis

Liver fixed in 10% of phosphate-buffered formalin (Polysciences, Warrington, PA, USA) was dehydrated with graded ethanol, embedded in wax (Paraplast Plus; McCormick Scientific, Richmond, IL, USA), sliced at 5 μm, mounted on slides, and oven-dried, and deparaffinized and subjected to H and E staining as previously described (Ganapathy-Kanniappan et al., 2012). To detect collagen deposition, the liver sections were stained using Sirius Red stain (PolySciences Inc. Warrington, PA, USA) or Masson's trichrome stain (Sigma Aldrich, St. Louis, MO) as per the instructions of suppliers. Quantification of collagen staining was performed using ImageJ software (National Institutes of Health, Bethesda, US) (Schneider et al., 2012). Staging of the fibrosis was performed according to the METAVIR scoring system in which on a 5-point scale, F0 denotes no fibrosis (normal) and F4 refers to advanced cirrhosis (Poynard et al., 1997). Further experiments were performed using the control (F0) and early phase (F1) fibrogenic liver.

### TaqMan Real-Time Polymerase Chain Reaction

Quantification of the fibrosis marker gene, collagen1alpha1 (*Col1*α*1*) was performed using TaqMan Universal Master Mix II with UNG (Applied Biosystems, MA, USA) in a Quant Studio 12K Flex Real-Time PCR System (Applied Biosystems) as described (Gajendiran et al., 2018). In brief, total RNA was extracted using the Trizol reagent (Thermo Fisher Scientific), followed by RNA clean-up (RNeasy kit, Qiagen Inc. Valencia, CA, USA) and subjected to reverse transcription using the High Capacity cDNA Reverse Transcription Kit (Applied Biosystems). Thus, the cDNAs synthesized were subjected to the real-time polymerase chain reaction (qPCR) using gene-specific Taqman probes, i.e., *Col1*α*1* (Probe ID; Mm00801666_g1) and β*2-microglobulin* (internal control, Probe ID: Mm00437762_m1) obtained from ThermoFisher Scientific Inc.

### ^3^H-TPP Liver Uptake and Biodistribution Studies

Liver uptake and tissue distribution of ^3^H-TPP in the respective groups (i.e., control and early phase, F1) were determined by tail vein injection of 370 kBq (10 μCi) of ^3^H-TPP (Min et al., 2004). Animals were euthanized 1 h post-injection, the blood was collected by cardiac puncture, and other organs were harvested for further analysis. All solid and liquid wastes of radioactive material were disposed of as per the institutional guidelines of the Radiation Safety Office. Quantification of the tissue uptake of ^3^H-TPP was performed as follows: a known quantity of tissue (e.g., 50 mg) was solubilized in the Solvable™ (Perkin Elmer Co., Waltham, MA, USA) as per the instructions of the supplier, followed by the addition of UltimaGold™ scintillation cocktail reagent (Perkin Elmer Co.), and the ^3^H was counted using a Beckman LS-6,500 liquid scintillation counter. Radioactivity determinations were normalized by the weight of the tissue and the amount of radioactivity injected, obtaining the percentage of injected dose/gram tissue (% ID/g) (Sands et al., 1994).

### Isolation of Mouse Liver Mitochondria

Mouse liver mitochondria were isolated as described (Rogers et al., 2011). The method of isolation is relevant for functional analysis such as the rate of respiration (oxygen consumption) using the Seahorse XF^96^ extracellular flux analyzer (Seahorse Bioscience, Billerica, MA, USA). Furthermore, the isolation procedure has been validated by several laboratories as well (Rogers et al., 2011; Iuso et al., 2017). In brief, mouse liver was extracted, rinsed with ice-cold phosphate-buffered saline to remove blood, and the liver was minced in ~10 volumes of ice-cold mito-isolation buffer (MIB), pH 7.2 [70 mM sucrose, 210 mM mannitol, 5 mM HEPES, 1 mM EGTA, and 0.5% (w/v) fatty acid-free BSA]. All subsequent steps of the preparation were performed on ice. The minced tissue was homogenized using a Glass/Teflon Potter Elvehjem homogenizer with not more than 10 strokes. Homogenate was centrifuged at 800 × *g* for 10 min at 4°C. Following centrifugation and careful removal of the lipid layer, the remaining supernatant was filtered through a double-layer cheesecloth into a separate tube and centrifuged at 8,000 × *g* for 10 min at 4°C. The supernatant was removed, the pellet containing the bulk of the mitochondria was resuspended in MIB, and the centrifugation was repeated. The final pellet was resuspended in a minimal volume of MIB. For functional assays, such as metabolic flux analysis, the mitochondria were used fresh and as quickly as possible. The rest of the mito-samples was stored at −80°C until further use. The protein concentration was determined using a 2D-Quant Kit (GE Healthcare, Piscataway, NJ, USA) (Ganapathy-Kanniappan et al., 2009).

### Immunoblotting

Immunoblotting was performed as described (Kunjithapatham et al., 2015). Control, F1 and F3 liver lysates, as well as the mitochondria isolated from the corresponding livers were subjected to relevant immune detection. In brief, a known quantity of the liver tissue was washed in PBS and homogenized in ice-cold RIPA lysis buffer containing protease and phosphatase inhibitor cocktails at 4°C using a Dounce homogenizer. The homogenates were centrifuged at 12,000 × *g* for 15 min at 4°C, the clear supernatants were collected, and the protein concentration was determined using a 2D-Quant Kit (GE Healthcare, Piscataway, NJ, USA). The samples were then resolved on a 4–12% Bis-Tris gel by electrophoresis with MOPS running buffer and blotted onto PVDF membranes (BioRad, Hercules, CA, USA) followed by immunoblotting with specific antibodies. Immune complexes were visualized by using the ECL-detection kit (GE Healthcare).

### Metabolic Flux Analysis

The mitochondria isolated from the mouse liver as mentioned above were used to determine the oxygen consumption rate (OCR) (Rogers et al., 2011; Iuso et al., 2017) using the Seahorse XF^96^ extracellular flux analyzer (Seahorse Bioscience, Billerica, MA, USA). In brief, an equal amount (~5 μg) of control and F1-mitochondria were plated into the Seahorse XFe^96^ assay plate, and all subsequent steps including the addition of substrate, reagents, and ADP and the duration of the assay were followed as described (Rogers et al., 2011). Unlike the cell-based assay, in this study, we used the organelle, mitochondria isolated from the respective livers. Thus, the absence of any cellular source renders the extracellular acidification rate (ECAR) inapplicable. Then, the MMP was determined based on the mito-uptake of Tetramethylrhodamine, methyl ester (TMRM) (Scaduto and Grotyohann, 1999). In brief, mitochondria (0.5 mg/ml) that were isolated from control and F1 and F3 livers were incubated with 5 mM glutamate, 5 mM malate, and 0.5 μM TMRM. Fluorescence at 546 and 573 nm excitation was monitored using an emission wavelength of 590 nm.

### Transmission Electron Microscopy

Control and fibrotic liver tissues were harvested and thoroughly rinsed in PBS buffer prior to the fixation. For mito-studies, the mitochondria were isolated from the respective mouse liver as described above. The liver and the corresponding mitochondria were fixed in 2.5% glutaraldehyde, 3 mM MgCl_2_, and 0.1 M cacodylate buffer, pH 7.2 overnight at 4°C. After buffer rinse, samples were post-fixed in 0.8% potassium ferrocyanide reduced 1% osmium tetroxide in buffer (1 h) on ice in the dark followed by 0.1 M sodium cacodylate buffer rinse. Samples were left at 4°C overnight in buffer, rinsed with 0.1 M maleate buffer, *En bloc* stained with 2% uranyl acetate (0.22 μm filtered, 1 h, dark) in 0.1 M maleate, dehydrated in a graded series of ethanol, and embedded in Eponate 12 (Ted Pella) resin. Samples were polymerized at 60°C overnight. Thin sections, 60–90 nm, were cut with a diamond knife on the Reichert-Jung Ultracut E ultramicrotome and picked up with Formvar coated copper slot grids. Grids were stained with 2% uranyl acetate in 50% methanol, followed by lead citrate, and observed using a Philips CM120 at 80 kV. Images were captured with an AMT XR80 high-resolution (16-bit) 8 Mpixel camera.

### Statistical Analysis

All data were analyzed using VassarStats (Lowry, 2004), and the difference between the two means was assessed by using either Mann–Whitney–Wilcoxon (MWW) test (unpaired) or the paired *t*-test as appropriate. The probabilities of *p* < 0.05 or as indicated were considered as significant.

## Results

### Staging Early and Advanced Liver Fibrosis in CCl_4_ Model

**Figure 1:**
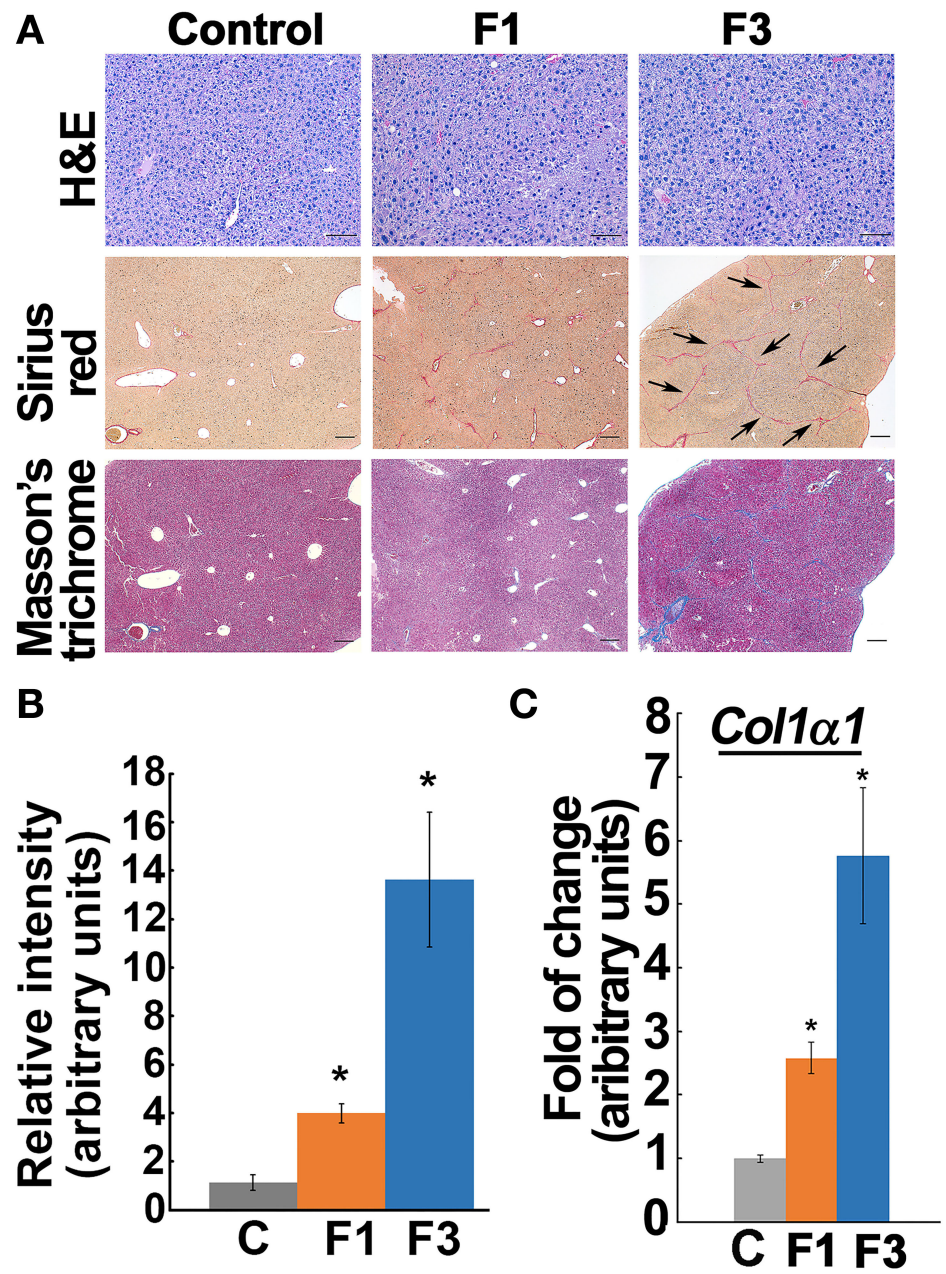
Staging early and advanced fibrosis in carbon tetrachloride (CCl_4_) model. **(A)** Histochemical staining of liver from control and experimental groups showed minimal collagen deposit, i.e., early fibrosis (F1), at 4 weeks of CCl_4_-administration. At 6 weeks, the bridging phenotype (indicated by arrows) and increased collagen deposition as verified by Sirius Red and Masson's trichrome indicate advanced fibrosis (F3). Based on the METAVIR scoring system, the 4 and 6 weeks of CCl_4_ administration correspond to F1 and F3 stages, respectively. Scale bar represents 100 μm in the Hand E staining and 200 μm in the Sirius Red and Masson's trichrome staining. **(B)** Bar graph shows quantification of collagen Sirius stain using ImageJ software. **(C)** *Col1*α*1* expression increased in the corresponding early (F1) and advanced (F3) fibrotic liver. Data represent mean ± SE (*n* = 5), *t*-test, *p < 0.05.

Histochemical staining by Sirius Red and Masson's trichrome for the fibrosis marker, “collagen deposition” demonstrated that 4 weeks of CCl_4_-exposure induced minimal fibrosis, i.e., collagen deposits without bridging, and corresponded to the F1-stage according to the METAVIR fibrosis score (Figures 1A,B, Supplementary Figure 1), whereas, liver from 6 weeks of CCl_4_-exposure led to increased collagen deposition and showed the characteristic “bridging fibrosis” corresponding to stage F3 (Figures 1A,B). Quantification of the *Col1*α*1* mRNA reflected the progression of fibrosis from early (F1) to advanced (F3) at 4 and 6 weeks of CCl_4_-administration (Figure 1C). ALT activity, one of the markers of liver function also showed a significant increase in F1 and F3 and corroborated the hepatic insult in CCl_4_-induced fibrogenesis (Supplementary Figure 2).

### ^3^H-TPP Liver Uptake and Biodistribution in CCl_4_ Model

**Figure 2:**
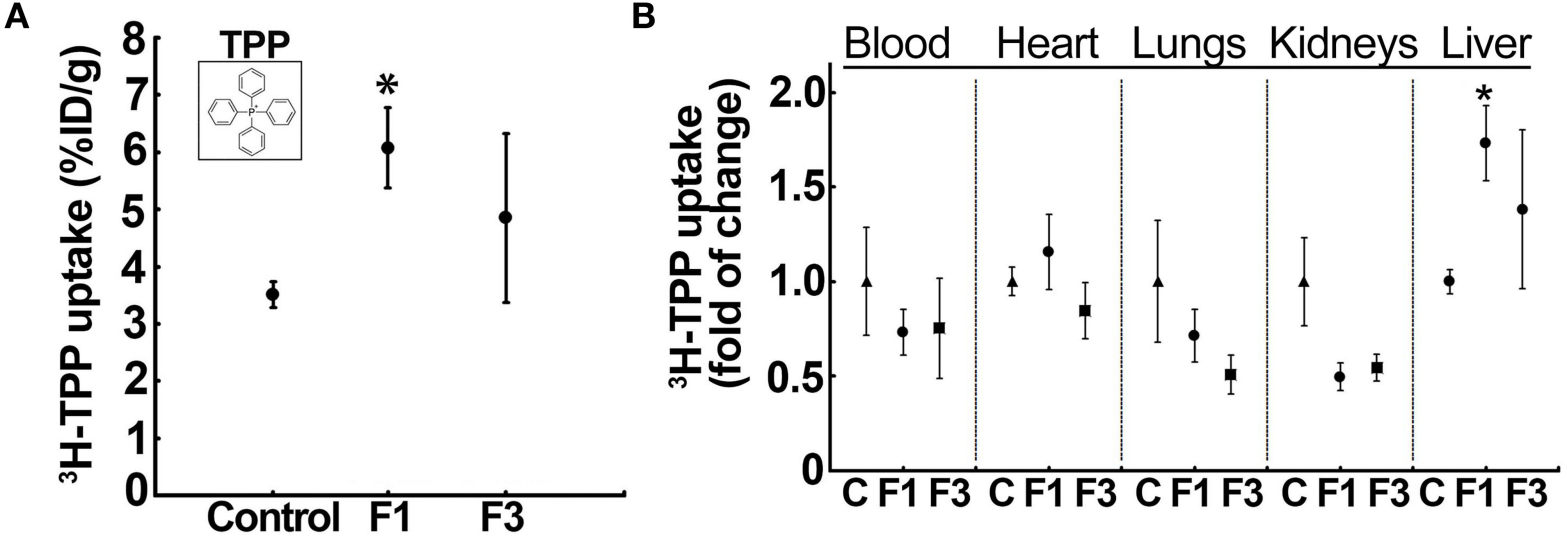
Liver uptake of the voltage-sensor, ^3^H-tetraphenylphosphonium (TPP), increases in early fibrosis. **(A)** ^3^H-TPP uptake (percentage of the injected dose, % ID) significantly increased in the F1 liver compared with the vehicle control and F3. Insert: chemical structure of TPP used as radiolabeled (^3^H)-TPP. Data represent mean ± SE (*n* = 5) MWW test (*p < 0.05). **(B)** Tissue distribution of ^3^H-TPP at F1 showed a significant increase in the liver uptake but not in other tissues, indicating hepatic fibrosis-related elevation in the net membrane potential of the liver. Data represent mean ± SE (*n* = 5). MWW test (*p < 0.05). Control, F1, early-stage fibrosis, F3, and advanced stage fibrosis. The vehicle (olive oil) controls of each batch (different weeks) were aggregated in a single group for analysis.

The liver uptake of ^3^H-TPP in mice indicated a significant increase in F1-liver (6.08 ± 0.69% ID) compared with the vehicle control (3.51 ± 0.23% ID/g) and F3-liver (4.85 ± 1.48% ID) (Figure 2A, Supplementary Figure 3A). Then, quantification of ^3^H-TPP-uptake by various organs between the control and F1 and F3 livers showed that only liver but not blood, heart, lungs, and kidneys demonstrate a statistically significant fold of increase in the accumulation of the voltage sensor, i.e., ^3^H-TPP (Figure 2B, Supplementary Figure 3B). The results show that the liver accumulation of ^3^H-TPP is significant in early fibrogenesis.

Noteworthy, ^3^H-TPP liver uptake significantly correlated with the serum ALT activity that showed a marked elevation in F1 (Supplementary Figure 4A). Then, the liver morphology as observed by transmission electron microscopy (TEM) corroborated the fibrotic phenotype of the F1 liver. Control liver showed normal morphology (e.g., subcellular organelles and glycogen granules) with no indicators of fibrogenesis (Supplementary Figure 4B). Conversely, CCl_4_-administered mice (F1) showed characteristic features of fibrosis such as the occurrence of multiple vesicles, fat droplets, presence of active Kupffer cells at the sinusoid with endothelial cell lining, and presence of collagen fibers (Supplementary Figure 4B).

### Liver Mito-OxPhos Capacity in CCl_4_ Model

**Figure 3:**
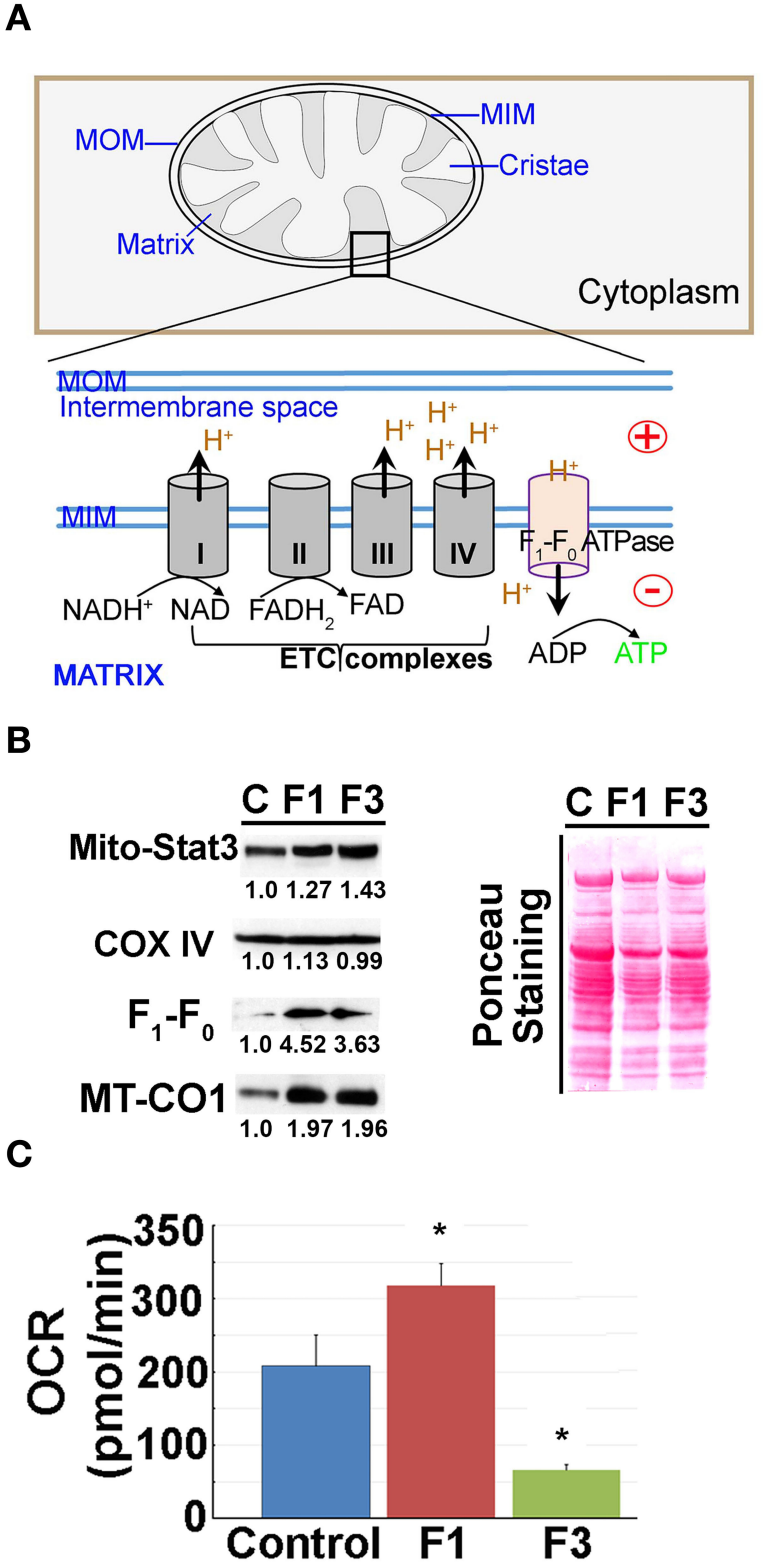
Mitochondrial (mito-) oxidative phosphorylation (OxPhos) upregulated in early fibrosis. **(A)** Schematic diagram showing the overview of the regulation of mito-membrane potential. In OxPhos, ETC complexes release protons (H^+^) into the space between the inner (MIM) and outer membranes (MOM) of mitochondria. H^+^ accumulation establishes an electrochemical gradient leading to a negative potential (–) in the matrix. Thus, a higher rate of OxPhos (e.g., ATP synthesis) results in increased negative potential in the mito-matrix. **(B)** Immunoblot of mito-proteins shows an increase in mito-translocation of STAT3, F_1_-F_0_ ATP synthase, and MT-CO1. COX IV remained unaltered. Immunoblot was re-probed for different targets to maintain the loading control. Numerical values at the bottom of the immunoblots represent the densitometry quantification of respective signals. Ponceau staining of the membrane shown for overall protein profile. **(C)** Metabolic flux analysis showing the oxygen consumption rate (OCR) of mitochondria isolated from the control, and F1 and F3 livers. Mitochondria showing a net increase in the OCR in the F1 liver. Unlike the typical cell-based assay, in this study, we used the mitochondria isolated from the respective livers as referred in the “Materials and methods” section. Hence, the extracellular acidification rate (ECAR) which is pertinent in the cellular assay is inapplicable. Data represent mean ± SE (*n* = 3), *t*-test (*p < 0.05).

**Figure 4:**
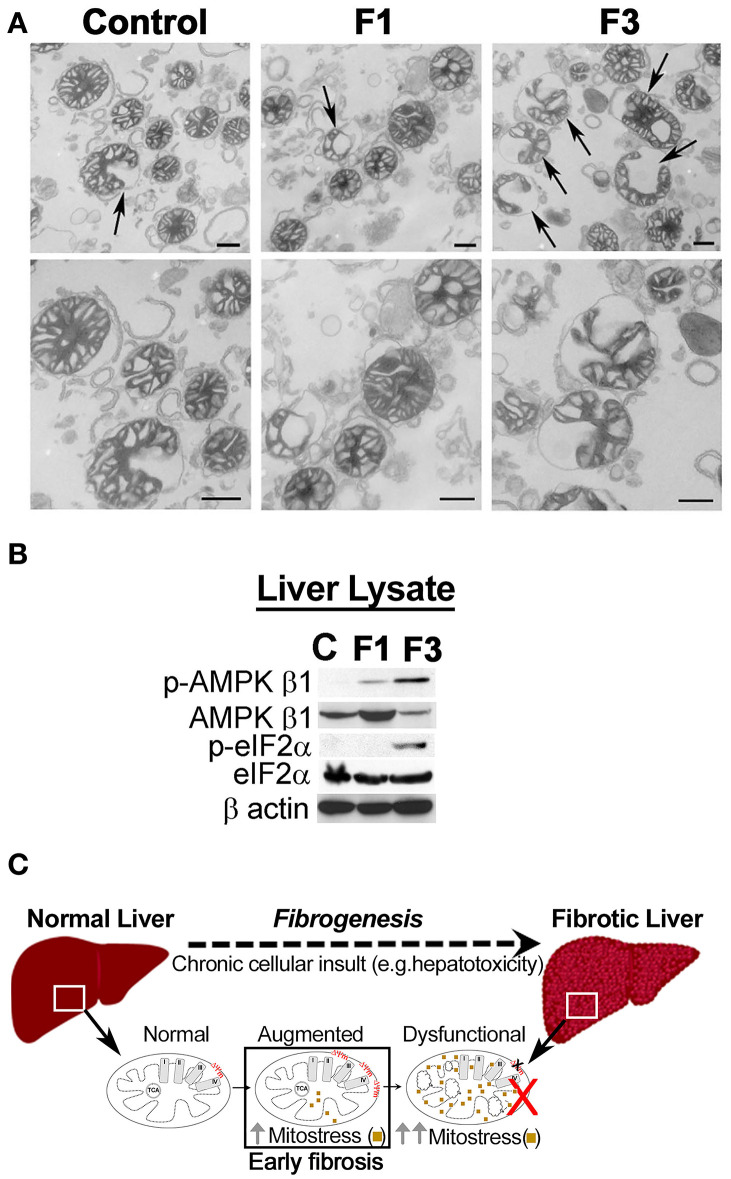
Mitochondria in fibrosis. **(A)** Transmission electron microscopy (TEM) observation of liver mitochondria in fibrosis. Mito-morphology in F1 remains similar to the control liver, whereas, in F3, mitochondria with altered morphology were prominent. Upper panel: arrows indicate variation in the appearance of mitochondria. Lower panel: magnification of the respective upper panel. Scale: 500 nm. (C) Control, F1, early, and F3, and advanced stages of fibrosis. **(B)** Immunoblot of the total liver protein shows CCl_4_-dependent induction of metabolic stress as indicated by the upregulation of p-AMPK β1 and p-eIF2α. β-actin is shown as the loading control. **(C)** Mito-response to hepatic insult in early fibrosis augments net voltage of the liver.

As critical determinants of membrane voltage, we then investigated the OxPhos/ETC enzymes (Figure 3A). Immunoblot analysis of mitochondria isolated from the control and F1 and F3 livers showed upregulation of ETC/OxPhos enzymes, F_1_-F_0_ ATP synthase, and MT-CO1 (Figure 3B). A concurrent increase in mito-translocation of STAT3 (regulator of OxPhos) implied fibrogenesis-related augmentation of OxPhos activity. Then, metabolic flux analysis of mitochondria isolated from the control and fibrotic livers showed distinctively elevated respiration (i.e., OCR) in F1 compared with the control (Figure 3C, Supplementary Figure 5). Intriguingly, OCR of F3 mitochondria diminished significantly (Figure 3C, Supplementary Figure 5). Further investigation of mito-uptake of TMRM, an indicator of MMP, also revealed an increase in TMRM uptake at F1 compared with control implying elevated mito-activity in the early stages of fibrosis (Supplementary Table 1). Mito-investigation by TEM *ex vivo* showed that the mito-morphology in early fibrosis remained intact and unaffected (F1) similar to the control, whereas, F3 liver showed more mitochondria with variations in appearance (Figure 4A). Corroborating the impaired mito-capacity in F3 and the level of *p*-AMPK and *p*-eIF2α, indicators of metabolic stress showed a marked elevation in the F3 liver (Figure 4B). Collectively, mito-metabolic response to hepatic insult augments the net voltage of the liver in early fibrosis (Figure 4C).

## Discussion

This study shows that the liver uptake of the membrane voltage sensor, ^3^H-TPP significantly increases in early fibrosis as confirmed by the onset of collagen deposition. Then, the upregulation of the OxPhos enzymes with a concomitant increase in mito-respiration in early fibrosis (F1) concurred with the augmented liver uptake of ^3^H-TPP. TPP has been implicated in the assessment pathophysiology of cancer (Min et al., 2004; Madar et al., 2007), cardiac disease (Higuchi et al., 2011; Gurm et al., 2012), and others, through the functional imaging modalities such as PET. However, its relevance in liver fibrosis/cirrhosis remains unknown, primarily due to the lack of any experimental study on the overall membrane voltage of the liver. Our findings provide the primary evidence that liver voltage may enable the detection of the fibrogenic liver at an early stage.

Mechanistically, the cellular uptake and the intramitochondrial accumulation of TPP depend on the electrochemical gradient across respective membranes, i.e., at cellular and mito-levels. The net positive/negative charge of the electrochemical gradient determines the membrane potential or the voltage. In principle, as cationic lipophilic agents, the intracellular entry of TPP is facilitated by the cellular/plasma membrane voltage which is generally −30 to −60 mV. Then, in metabolically active mitochondria, consequential to the functions such as the OxPhos/ETC, and/or increase in the total mito-matrix volume, the MMP remains increased (−150 to −180mV). Such an elevated MMP enables 100× to 500× more mito-accumulation of the lipophilic cations (e.g., TPP and TMRM fluorescent dye) (Murphy, 2008; Zielonka et al., 2017). Conversely, the TPP or TMRM uptake is reduced or decreased in cells with diminished MMP, i.e., membrane-depolarization, underscoring the role of MMP in TPP uptake. Accordingly, the propensity of TPP to accumulate in cells with higher MMP is exploited for the therapeutic targeting of mitochondria in preclinical investigations (Han et al., 2014; Pathak et al., 2014; Marrache and Dhar, 2015). Collectively, the plasma membrane voltage, mito-matrix volume, and membrane potential impact the rate of TPP uptake. Nevertheless, our findings from the CCl_4_ (hepatotoxicity)-induced model of fibrogenesis indicate that unlike the advanced fibrosis (Krahenbuhl et al., 2000; Rodrigues and Steer, 2000; Sun and Kisseleva, 2015), liver-mitochondria of the early phase of hepatic insult exhibit augmented respiration/ETC activity indicating the involvement of MMP in the increased liver accumulation of TPP.

In addition, multiple lines of evidence indicate that hepatotoxicity-induced apoptosis may promote cell proliferation and the associated mito-functions, as a compensatory mechanism, at least in the initial stages (Mehendale et al., 1994; Trost and Lemasters, 1997; Lemasters and Holmuhamedov, 2006; Jones et al., 2010; Bajt et al., 2011; Rehman et al., 2016; Wimborne et al., 2020). Since mito-function is an integral part of cell growth and proliferation, it is plausible that the overall increase in MMP is perhaps a result of increased mito-biogenesis, mito-activity, and/or the cell number. However, our data on the mito-respiration, a functional assay (which used an equal quantity of mitochondria), revealed an enhanced rate of oxygen consumption (under normalized mito-content) compared with the control, indicative of augmented mito-activity.

Then, several elegant reviews have underscored the role of STATs, particularly STAT3 in liver pathophysiology (Kong et al., 2012; Yang and Rincon, 2016). Transcriptionally, STAT3 promotes aerobic glycolysis and downregulates OxPhos. Conversely, its translocation to mitochondria preserves mito-function, upregulates OxPhos, and promotes survival (Poli and Camporeale, 2015). In fact, STAT3 regulates the functional efficiency of ETC complexes suggesting its critical role in cellular respiration (Wegrzyn et al., 2009). Intriguingly, in liver pathology, STAT3 plays two distinctive roles depending on the hepatic cell type; in hepatocytes, it is cytoprotective, whereas, in the HSCs, it is profibrogenic (Kong et al., 2012). Thus, an increase in mito-translocation of STAT3 as a mito-stress indicator and concurrent upregulation of F_1_-F_0_ ATP synthase and MT-CO1 in F1 implies a mito-metabolic stress response in early fibrogenesis. It remains to be known whether the enhanced uptake of TPP in fibrogenesis is relevant in other fibrogenic conditions (e.g., viral infection, biliary fibrosis) or limited to chemically (CCl_4_)-induced fibrosis.

Liver fibrosis and cirrhosis are pathological sequelae for several chronic liver diseases that may eventually lead to death, hence early diagnosis is critical for the management of the disease (Farazi and DePinho, 2006; Hernandez-Gea and Friedman, 2011). Despite significant progress in other imaging modalities (Tapper and Loomba, 2018), such as MR elastography (MRE) and transient elastography (TE), that rely on the elasticity/stiffness of the fibrotic/cirrhotic liver, the potential of PET diagnostic-imaging of liver fibrosis remains obscure. In this study, we reported an experimental study for a new PET-relevant functional marker that may have implications in the early diagnosis of liver fibrosis.

The diagnostic/therapeutic potential of membrane voltage has been recognized in human diseases (e.g., cancer and cardiac disease) (Min et al., 2004; Madar et al., 2007; Higuchi et al., 2011; Gurm et al., 2012; Rehman et al., 2016), hence understanding the net liver-voltage during the initial phases of fibrogenesis may have clinical implications. Mito-membrane voltage-dependent PET imaging relies on the difference in membrane voltage between normal and abnormal cells/tissues. However, in liver fibrosis/cirrhosis, until now, there is no known functional marker or a molecular mechanism that is applicable or relevant for voltage-dependent PET-imaging. In this study, we reported a potential PET-relevant functional marker that on further validation may have implications in the early diagnosis of liver fibrosis. Nevertheless, it remains to be determined whether the ~2-fold increase in the fibrotic-liver uptake of ^3^H-TPP in our mouse model could still be exploited in the functional imaging to distinguish fibrosis. Future studies using clinically relevant probes are underway to delineate the significance and translational potential of mito-membrane voltage-dependent PET imaging in the early detection of liver fibrosis.

## Data Availability Statement

The original contributions presented in the study are included in the article/Supplementary Material, further inquiries can be directed to the corresponding authors.

## Ethics Statement

The animal study was reviewed and approved by Institutional Animal Care and Use Committee (IACUC), Johns Hopkins University School of Medicine.

## Author Contributions

EM and SG-K conceived this study and wrote the manuscript. SG-K designed the experiments, performed mito-isolation, functional experiments, and data analysis. HP and SG-K performed *in vivo* investigation, histochemical staining, and TEM studies. All authors contributed to the article and approved the submitted version.

## Conflict of Interest

The authors declare that the research was conducted in the absence of any commercial or financial relationships that could be construed as a potential conflict of interest.

## Publisher's Note

All claims expressed in this article are solely those of the authors and do not necessarily represent those of their affiliated organizations, or those of the publisher, the editors and the reviewers. Any product that may be evaluated in this article, or claim that may be made by its manufacturer, is not guaranteed or endorsed by the publisher.
