# Erratum: Augmented Liver Uptake of the Membrane Voltage Sensor Tetraphenylphosphonium Distinguishes Early Fibrosis in a Mouse Model

**DOI:** 10.3389/fphys.2021.830151

**Published:** 2021-12-22

**Authors:** Frontiers Production Office

**Affiliations:** Frontiers Media SA, Lausanne, Switzerland

**Keywords:** liver fibrosis, membrane-voltage sensor, tetraphenylphosphonium (TPP), mitochondrial respiration, electron transport chain, carbon tetrachloride, liver voltage

Due to a production error, the following words were misspelled: “Fibro genic liver” in the Materials and Methods and Discussion sections should be “fibrogenic liver”; and “Fibro genesis” in the Materials and Methods section should be “fibrogenesis.”

A correction has been made to the **MATERIALS AND METHODS** section, subsection **Histopathology and Staging Fibrosis**:

“Liver fixed in 10% of phosphate-buffered formalin (Polysciences, Warrington, PA, USA) was dehydrated with graded ethanol, embedded in wax (Paraplast Plus; McCormick Scientific, Richmond, IL, USA), sliced at 5 μm, mounted on slides, and oven-dried, and deparaffinized and subjected to H and E staining as previously described (Ganapathy-Kanniappan et al., 2012). To detect collagen deposition, the liver sections were stained using Sirius Red stain (PolySciences Inc. Warrington, PA, USA) or Masson's trichrome stain (Sigma Aldrich, St. Louis, MO) as per the instructions of suppliers. Quantification of collagen staining was performed using ImageJ software (National Institutes of Health, Bethesda, US) (Schneider et al., 2012). Staging of the fibrosis was performed according to the METAVIR scoring system in which on a 5-point scale, F0 denotes no fibrosis (normal) and F4 refers to advanced cirrhosis (Poynard et al., 1997). Further experiments were performed using the control (F0) and early phase (F1) fibrogenic liver.”

A correction has been made to the **DISCUSSION** section, first paragraph:

“This study shows that the liver uptake of the membrane voltage sensor, ^3^H-TPP significantly increases in early fibrosis as confirmed by the onset of collagen deposition. Then, the upregulation of the OxPhos enzymes with a concomitant increase in mito-respiration in early fibrosis (F1) concurred with the augmented liver uptake of ^3^H-TPP. TPP has been implicated in the assessment pathophysiology of cancer (Min et al., 2004; Madar et al., 2007), cardiac disease (Higuchi et al., 2011; Gurm et al., 2012), and others, through the functional imaging modalities such as PET. However, its relevance in liver fibrosis/cirrhosis remains unknown, primarily due to the lack of any experimental study on the overall membrane voltage of the liver. Our findings provide the primary evidence that liver voltage may enable the detection of the fibrogenic liver at an early stage.”

A correction has been made to the **MATERIALS AND METHODS** section, subsection Fibrogenesis by CCl_4_:

“Animal experiments were performed as approved by the Institutional Animal Care and Use Committee. To establish the liver fibrosis model, 3–4 week old male C57BL/6 mice (15–20 g body weight) were procured from the Charles River Laboratories Inc. (Wilmington, MA, USA) and maintained in a temperature-controlled room with an alternating 12-h dark and light cycle. To determine the fibrotic stage, mice were randomly divided into control (vehicle, *n* = 7) and experimental groups (*n* = 7) representing 2, 4, and 6 weeks of CCl_4_ administration. Fibrogenesis was induced by intraperitoneal administration of 20% solution of CCl_4_ (Sigma Chemical Co., St. Louis, MO, USA) in olive oil (vehicle) (Mehendale et al., 1994; Wang et al., 2007; Jin et al., 2011; Karthikeyan et al., 2016), at a dose of 0.5 μl/g bodyweight every week thrice for up to 6 weeks. Histopathology and analysis of fibrosis markers were used to determine the early fibrotic stage.”

The publisher apologizes for this mistake. The original version of this article has been updated.

## Publisher's Note

All claims expressed in this article are solely those of the authors and do not necessarily represent those of their affiliated organizations, or those of the publisher, the editors and the reviewers. Any product that may be evaluated in this article, or claim that may be made by its manufacturer, is not guaranteed or endorsed by the publisher.
