# Supplementary material for: Augmented Liver Uptake of the Membrane Voltage Sensor Tetraphenylphosphonium Distinguishes Early Fibrosis in a Mouse Model
Source: Front Physiol. 2021 Oct 25;12:676722. doi: 10.3389/fphys.2021.676722 (PMC8573124; doi:10.3389/fphys.2021.676722)
Supplement: Supplementary file 1 [file Data_Sheet_1.pdf]

SUPPLEMENTARY MATERIAL

Supplemental data include five figures and one table.

Figure S1. Staging early and advanced fibrosis in CCl_4 model. Histochemical staining of liver from control, and experimental groups showed the earliest occurrence of collagen deposits (i.e.) early fibrosis (F1), at 4-weeks of CCl_4-administration. At 6-weeks, the bridging phenotype (indicated by arrows) and increased collagen deposition as verified by Sirius red and Masson's trichrome indicate advanced fibrosis (F3). Whereas at 2-weeks of CCl_4-administration there was no detectable collagen deposits. Hence, for subsequent investigations, we used groups 4- and 6-weeks of CCl_4 administration representing F1 and F3 correspondingly, according to METAVIR scoring system. Scale bar represents 100 µm in the H&E staining and 200 µm in the Sirius red staining.

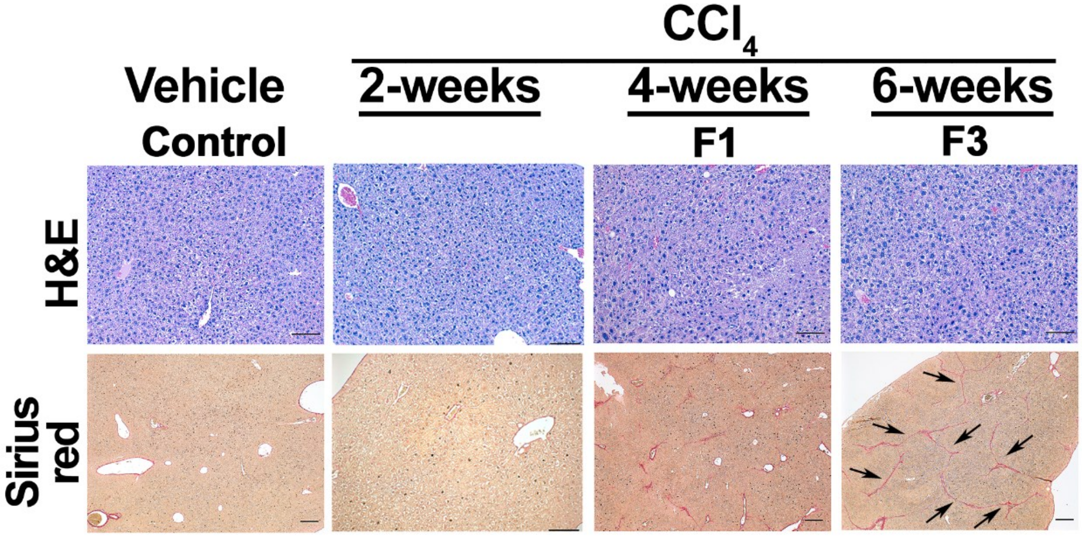

Figure S2. Increase in serum ALT activity at 4-weeks (F1) ad 6-weeks (F3) of fibrogenic induction by CCl_4 in a mouse model. (*, t-test).

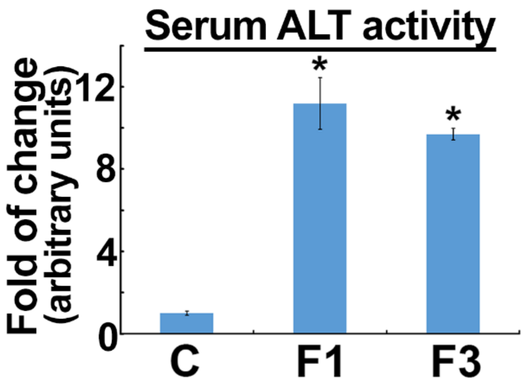

Figure S3. ^{3}H-TPP uptake and tissue distribution in a mouse model of CCl_4-induced hepatic fibrosis. **A**. ^{3}H-TPP accumulation (percentage of the injected dose, % ID) in the liver significantly increased at 4-weeks ((6.08±0.69 %ID) (*, $p<0.05$)) and 6-weeks ((4.85±1.48 %ID) ($p\leq0.05$)) of CCl_4-exposure compared to the vehicle control (3.51±0.23 %ID/g). Note, the vehicle (olive oil) controls of each batch (different weeks) were aggregated in a single group for analysis. **B**. Biodistribution of ^{3}H-TPP in CCl_4-induced mouse model of liver fibrosis. The overall accumulation of ^{3}H-TPP in the blood remained very low in the control as well as various groups of fibrogenesis. Heart, Lungs and Kidneys showed an overall higher accumulation of ^{3}H-TPP in the control, and different groups of fibrogenesis, but no significant association liver fibrogenesis, particularly with the early (4-weeks) and advanced fibrosis (6-weeks). Data represent mean +/- SE.

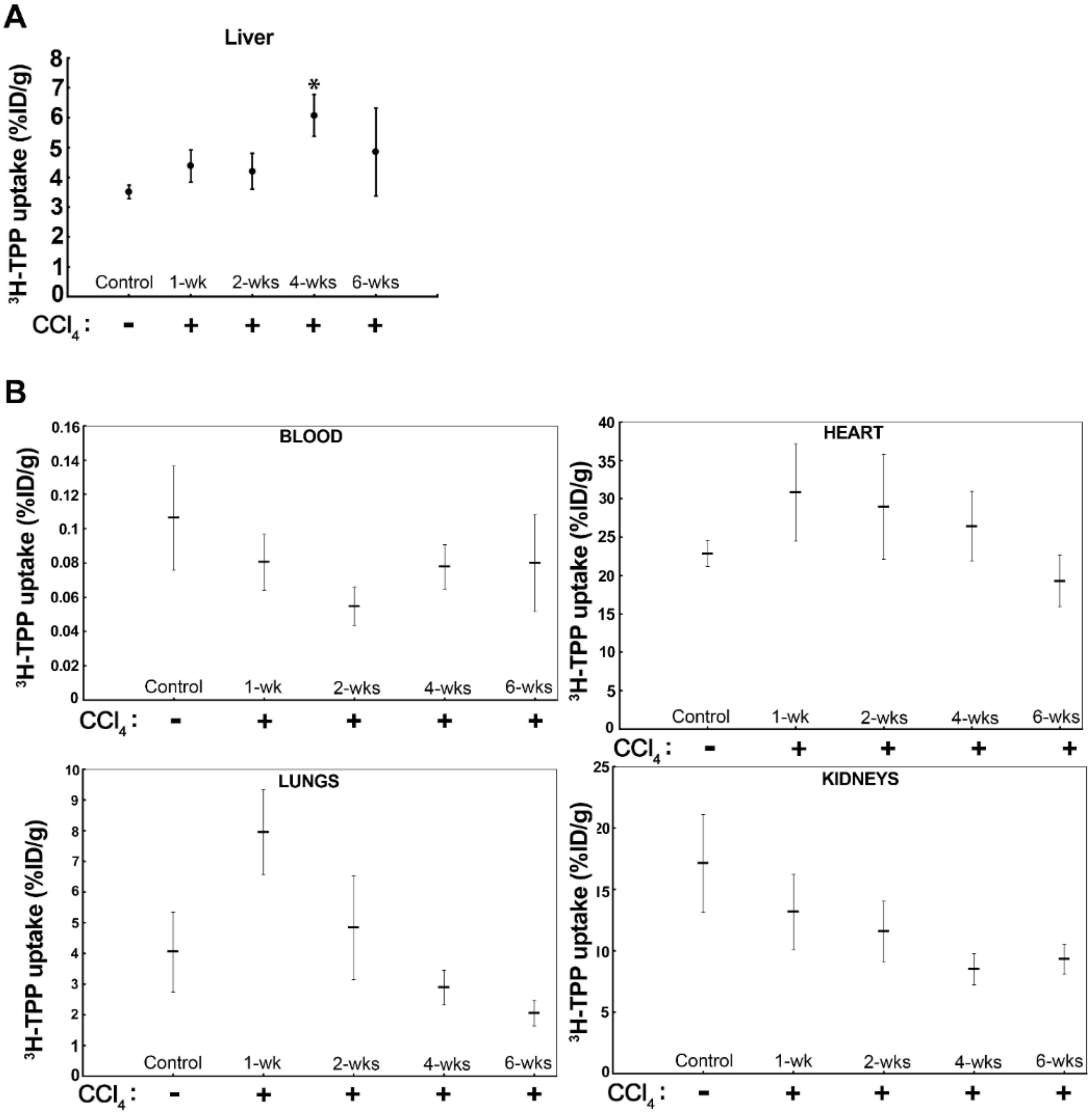

Figure S4. Elevated liver uptake of ^{3}H-TPP correspond to early fibrosis. **A**. The elevated serum ALT activity (line diagram) correlates with the liver uptake of ^{3}H-TPP (bar graph) in early fibrosis. *, p< 0.05; **, p<0.01. **B**. Electron micrographs of corresponding liver sections (control and F1) showing fibrogenic characteristics at early fibrosis. Col, collagen fiber; En, endothelial cells/lining; Er, endoplasmic reticulum; F, fat droplet; Gly, glycogen granules; Kc, Kupffer cell; Mt, mitochondria; N, nucleus; V, vesicle.

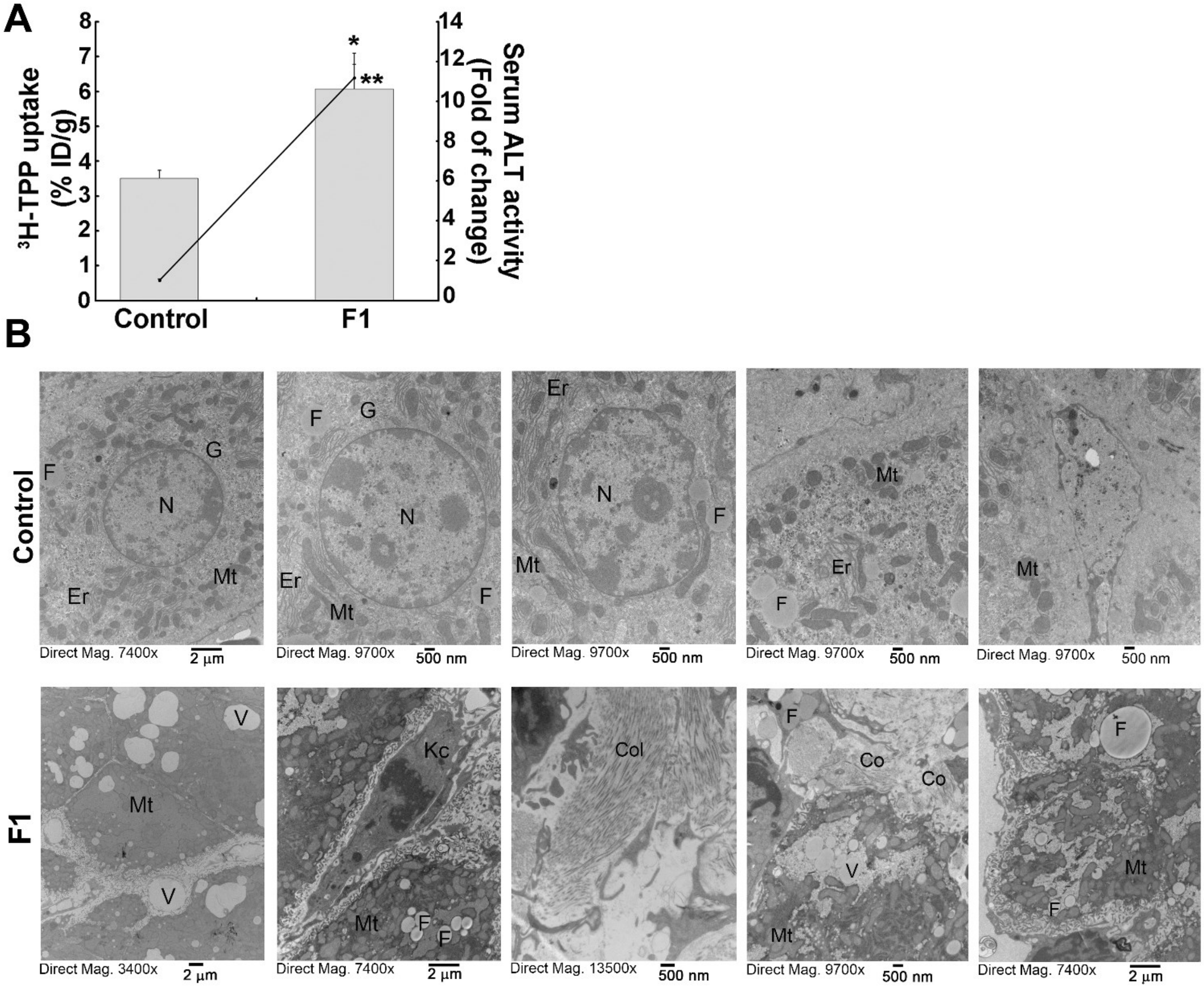

Figure S5. Metabolic flux analysis showing the oxygen consumption rate of mitochondria isolated from the control, F1 and F3 liver. Mitochondria showing a net increase in the oxygen consumption rate (OCR) in F1 liver. Note, unlike the typical cell-based assay, here we used mitochondria isolated from the respective livers as referred in the materials and methods section. Although, in an ideal condition, the FCCP would demonstrate a higher oxygen uptake, noteworthy, it depends on the absolute quantity of mitochondria. As indicated by the manufacturer's Application Note (Seahorse Bioscience, Billerica, MA, USA), there is a NON-LINEAR relationship between the mito-quantity and OCR/FCCP. Thus, the elevation in OCR in ADP is sufficient to demonstrate CCl_4-dependent changes in mitochondrial respiration. Nonetheless, in our experiment we used the same quantity of mitochondria for the control and experimental groups rendering the comparison of OCR between the respective mitochondria as rational to demonstrate the increased rate of electron transport chain (ETC) activity which is further substantiated by immunoblot data (upregulation of the F_1-F_0 ATPase, Fig. 3B).

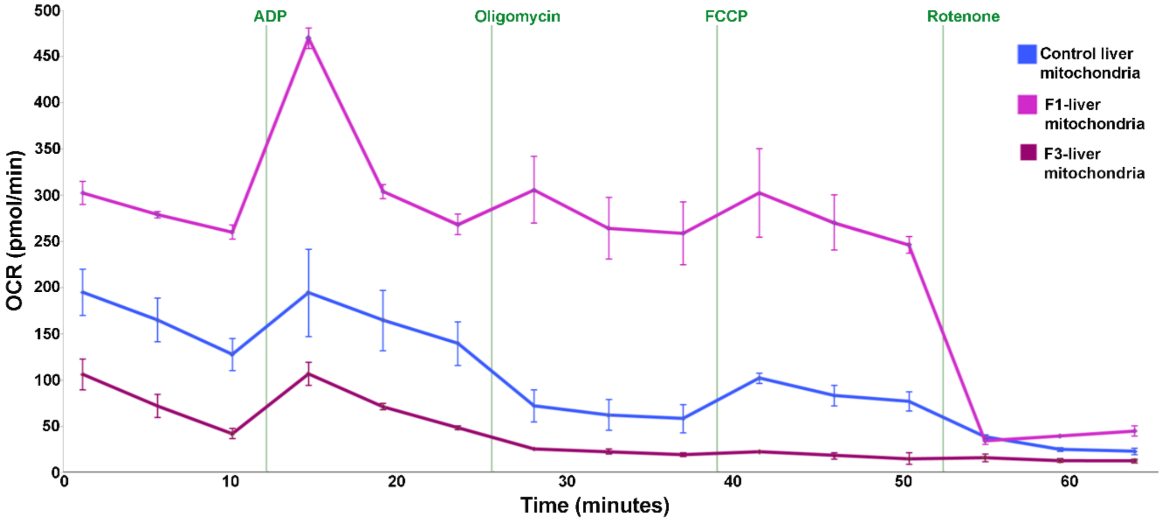

Supplementary Table-1: TMRM uptake by mitochondria isolated from control, F1 and F3 liver. Data represent mean ± SE of replicates (n=3). *, $p<0.05$.

	Control	F1 *	F3
**TMRM uptake** (arbitrary units)	0.049262 ± 0.000191	0.066639 ± 0.000157	0.043613 ± 0.000224
